# What Can Affective Science Contribute to Eradicating Structural Racism?

**DOI:** 10.1007/s42761-022-00110-z

**Published:** 2022-03-22

**Authors:** Lasana T. Harris

**Affiliations:** Department of Experimental Psychology, University College London, 26 Bedford Way, WC1H 0AP, London, United Kingdom

**Keywords:** affective science, structural racism, diversity science, inclusivity

## Abstract

This introduction to the Special Issue in *Affective Science* on structural racism lays a challenge to affective science researchers: improve the inefficiency in our science. It describes how structural racism leads to inefficiencies in idea generation, technological development, training, and career progression in our science, limiting its ability to fully discover the role of affect in the human condition. It briefly describes the content of the special issue, and attempts to start a dialogue about best practices for inclusive science.

Structural racism^1^ is a discriminatory behavior manifested in structures with a history of perpetuating, supporting, and enabling oppression, or failing to protect from oppression, a group of racialized people. This definition makes structures the key concept, and includes institutions, systems, organizations, pedagogy, and ideology. These structures facilitate experiences of racism and continued discrimination against racialized people, specifically people of color, broadly defined. How does structural racism affect us as human beings constituting the academic field affective science? And how can our research contribute to eradicating structural racism?

Structural racism leads to inefficient affective science. In the past, science has risen to inefficiencies in its system and self-corrected in the pursuit of truth and knowledge. Similarly, we must course-correct as affective scientists and address inefficiencies resulting from structural racism with solutions targeting how we conduct our science. We have faced and met similar challenges around the replication crisis, providing solutions that improved our science (see Open Science Collaboration, 2015); similar improvement can be made to continue to eradicate racism from our academic field.

Stated differently, as affective scientists, our science needs to improve to better address systemic problems like racism. This requires not simply the technological and theoretical advances that provide further insight, but recognizing humanity across the globe, and gearing our technology and theory with all human beings in mind. As a field, we are missing vast amounts of data about actual human beings when we just conduct experiments in Western, Educated, Industrialized, Rich, and Democratic (WEIRD) societies (Henrich et al., 2010). A lack of diversity in our scientists restricts the research questions we posit as a field, limiting our theoretical and technological breadth. Development of technology that only caters to people with one characteristic uncommon in wide swaths of humanity limits our insight into human behavior and affective processes. Poor terminology reinforces cultural narratives about group differences.

Moreover, we must consider the impact of structural racism on researchers who are racialized. Being of predominantly non-European descent makes life difficult as an academic. Negative feedback, endemic in academia, is difficult to interpret. Was I not selected because of my perceived race, or because there are flaws in my research program? Was I only selected because of my perceived race, not the strength of my scholarship? Such attributional ambiguity eternally haunts racialized people, making improvement difficult since feedback can never fully be trusted (Crocker et al., 1991; Mendoza-Denton et al., 2010), leading to real deleterious physiological consequences (Mendes et al., 2008). Moreover, most racialized people are on a novel path, with few if any role models and mentors to turn to who can truly understand their unique journey and the challenges faced. Additionally, there exists an inordinate burden not simply to serve on diversity-related committees and weigh in on such matters, but to be represented as a person of color so that the organization, committee, etc. does not appear as White. Such demands exponentially increase workload, making it difficult to progress at the same rate as colleagues without such burdens, all the while striving under a perception of inferiority simply because such people do not populate academia. These concerns were not addressed in this Special Issue but are features of the experience for many of our colleagues. Post George Floyd’s murder and the resurgent Black Lives Matter movement, we need to turn our attention to our own institutions, practices, and ways of working as a field and improve the inefficiency in our own corners of academia where we can exert some influence. This will also improve the quality of our science, ensuring that novel, innovative approaches continue to bubble up from people with very diverse experiences of what it is to be human.

## Special Issue Content

We are delighted to publish five articles in this special issue. In all the articles, we have tried to model best practice for inclusive science. All articles acknowledge WEIRD limitations in science. We have taken great pains to ensure that each article has used language in the most objective, scientific fashion, particularly when describing people of predominantly African descent. For instance, we use terms to describe people that acknowledge their humanity, describing them scientifically as more than a category label by noting where the sample was collected, such as Black American people, or African American people. This is also good non-WEIRD practice that limits inferences to similar cultural contexts. Moreover, we have ensured that traits attributed to such people are attributed to their characteristics, rather than the people themselves. This should reduce the othering of such people, and highlight the psychological phenomena that are under examination. There may be nomenclature or other failures still present in these articles that are realized in hindsight; however, we did our best to ensure that the articles are written in a culturally sensitive way, and that WEIRD gaps are highlighted and acknowledged.

Language is addressed in the first commentary that explores linguistic racism, and its impact on African American people. It argues that such racism promotes code-switching as a protective mechanism that depends on mentalizing in service of impression management, bounded by available cognitive resources. As such, code-switching is cognitively costly, putting the person at a disadvantage. Crucially, stereotype threat—a threat that occurs once one’s social identity is made salient—is identified as the affective trigger. It makes recommendations for all of us as a field to better recognize the heterogeneity of the field, reducing environments that make race salient and promote code-switching, and refine our measurement to be more sensitive to heterogeneous languages.

The second commentary takes the issue of measurement further and considers how one technology in particular electroencephalography (EEG)- is limited in the populations it can be used to study. Because EEG requires electrodes contacting the scalp, and many traditional hairstyles in people of predominantly African descent require that their tightly coiled hair is packed in more robust strands tightly bound to the head, then it is impossible to use a typical EEG cap with populations sporting such hairstyles. This consequence of structural racism nicely highlights the systemic inefficiency in our field, and the commentary discusses bioengineered solutions that consider the variety of human beings and their hair.

The final commentary again looks at problems inherent to our paradigms, this time considering how structural bias affects our theorizing about emotion reasoning. It highlights how considering a single cultural or racial perspective when considering reasoning development contributes to a scientific norm centered on that perspective and ignores the perspective of traditionally marginalized groups. It explores this specifically in the American context, looking at Black and White American people’s continued interracial animosity.

We go beyond the USA when considering empirical work that addresses structural bias. Our first empirical article uses the COVID-19 pandemic as a natural intervention to explore feelings of white guilt; it demonstrates reduced endorsement of this sentiment during the pandemic relative to before. Such work highlights how real-world events impact structural racism and can elucidate mechanisms that can become levers for societal change.

We also present experimental work showing that machine learning approaches to facial expressions of emotions are more likely to categorize European American male faces as angry, not African American faces, inconsistent with common affective science operationalizations and cultural stereotypes. This simultaneous demonstration of the inefficiency of our science and function of our brain emphasizes the powerful influence of structural racism in our academic field and society.

Our final empirical article considers an anti-racist approach to measuring emotion knowledge in early childhood. It describes initial development of a novel measure of emotion knowledge by attending to the race and ethnicity of the stimuli, translating appropriately to facilitate performance from Latinx children, and testing the measure in an ethnically and linguistically diverse sample. This demonstration of an approach to measurement models best practice for affective science.

## Personal Reflections

One of the points I advocate for above is a full recognition of humanity in science. As people studying something as critical to human behavior as affect, we must be aware of how our personal experiences, values, and beliefs influence the science we conduct. As part of this tradition, and given the nature of this topic, I deviate from previous editorial norms to describe my own reflections on structural racism as a racialized person. I begin with a brief and mostly benign glimpse of my experience with structural racism. When we first launched the call for this special issue in *Affective Science*, I received an email from a researcher asking if we could define structural racism. Though the question itself was not inappropriate, the email contained only ‘could you define structural racism?’ This seemed a departure from the traditional email sent to an editor at a journal; there was no greeting, faux pleasantries, or any of the other norms common in academic communication. Without evidence to ascertain whether this was indeed true, I had only my own experiences to serve as anecdotes. Given my background as not only a person of color, but someone who was born and raised in a non-WEIRD society, I granted myself permission to label the email as a micro-aggression (Pierce, 1970, 1974), a symptom of precisely the phenomenon at hand in this special issue. As I did in my social role as journal editor in response to the request for definition, I defined structural racism in my polite response to the email as I did at the beginning.

People are at the heart of structures that perpetuate racism, including academic fields, societies, and institutions. Eradiction requires an understanding of how people perpetuate these behaviors, often without conscious awareness. Moreover, since affect drives behavior (Frijda, 1988), affective science has the ability to contribute to our understanding, amelioration, and eventual eradication of structural racism.

One place we can begin to make this course correction surrounds the terms we use to describe the human beings we study. I personally, as someone of predominantly African descent, am not in favor of the term ‘Black’ since it conjures Manichean associations to moral badness or darkness (Harrell, 1999). It is also juxtaposed with the term ‘White’ that culturally stands for purity, moral goodness, and light. Skin color is heterogeneous within racial and ethnic groups, and there is no genetic basis to racial group differences; they are social constructs (Rutherford, 2020). Moreover, few people, particularly those in the New World contexts, can claim to genuinely be descendant just from a single race (Fortes-Lima & Verdu, 2021). Thus, these terms have social and political meanings, not scientific significance, and their use in science can promote prejudice and discrimination (Hall et al., 2021). I consider myself first and foremost a scientist. I acknowledge that this is my opinion, not one shared by the field, or other people of predominantly African descent, but I raise it to highlight both the heterogeneity of the experience that can lead to alternate points of view, but also to start a discussion about how we choose labels to describe the focus of our research: human beings.

We use conventions from culture, but in so doing, we fix our science within these cultural representations, making it more likely that features of structural biases affect the quality of our science. Given that we are studying human beings, these social meanings should be accounted for, but as a field, we perpetuate the harmful effects of such categorizations when using them in scientific reports to describe people. Indeed, cutting edge scientific research has been historically used to support a dehumanized perception of African people, justifying their enslavement and denigration (Eberhardt, 2020). We must do better. I believe we can do better and improve our science.

Despite its brevity, I have written this editorial, and we have assembled that Special Issue, over many months. I have returned to it when a situation arises or time allows in order to document the occurrence, or to simply have an outlet for the resulting frustrations. In so doing, it has been a cathartic experience, but also infuriating, because the fact that I must write this instead of a review, empirical or theoretical paper, or grant, just reminds me of the problems this issue attempts to address. I hope you find the research, ideas, and opinions in it useful, and I hope you feel motivated to address structural racism in affective science.

## Footnotes

1 I consider structural and systemic racism as synonyms, therefore use just the former throughout.
